# *CMJ* joins the call of health journals for immediate actions on global environmental crisis

**DOI:** 10.3325/cmj.2021.62.433

**Published:** 2021-10

**Authors:** Svjetlana Kalanj-Bognar, Anton Glasnović

**Affiliations:** *Croatian Medical Journal*, Zagreb, Croatia *svjetlana.kalanj.bognar@cmj.hr*

In early September 2021, more than 200 medical journals worldwide simultaneously published an editorial dealing with the dramatic impact of global warming on Earth’s ecosystems and human health (1). The editorial called for immediate concerted actions of policy-makers, especially health professionals, emphasizing their role in the efforts to prevent/alleviate further environmental harms. It was made publicly available ahead of three crucial events: the UN General Assembly meeting, UN Biodiversity Conference in Kunming, and the COP 26 UN Climate Change Conference in Glasgow.

Research on previous abrupt climate changes in Earth’s history has demonstrated beyond any doubt that the most recent change, characterized by a rise in average surface temperature and atmospheric carbon dioxide emissions compared with the pre-industrial period, has been caused mostly by human activities and to a lesser degree by natural factors. The understanding of complex systems such as Earth’s climate has been significantly advanced by the research of Syukuro Manabe, Klaus Hasselmann, and Giorgio Parisi, winners of the 2021 Nobel Prize in physics, who created a model for quantification and prediction of climate changes (2). Another Nobel laureate, atmospheric chemist Paul J. Crutzen, coined the term Anthropocene to describe the relatively short historical period in which humans profoundly affected the environment. The Anthropocene is considered to have started in 1950s, when the first nuclear bomb tests left permanent traces of human activities on Earth (3). The scientific community still discusses the need to introduce a new geological era, but the arguments in favor of the introduction stem from the fact that in only 70 years humans have changed the atmosphere and environment to a degree that would have been possible to occur naturally in millions of years.

The most significant climate changes, recorded in the last decade, have been happening in a world fraught with political and economic divisions, in an unsustainable economic system detrimental for both biological systems and human life. Having this in mind, the editorial that unified a remarkably large number of medical journals strongly conveys the message of personal and social responsibility of health professionals to contribute to the creation of global policies that will hopefully lead to a “sustainable, fairer, resilient, and healthier world.” Due to a highly disproportionate wealth distribution between countries, the concept of a fair and healthy world for everyone is unfortunately far from being realized. This concept has been additionally compromised during the COVID-19 pandemic, which caused an unprecedented global health crisis and brought to light huge inequalities in health care availability. Thus, the need for global actions to be taken based on the principles of solidarity and equity is underlined by the editorial’s subtitle – “Wealthy nations must do much more, much faster.”

The initiative launching the “Call for Emergency Action to Limit Global Temperature Increases, Restore Biodiversity, and Protect Health” has been also recognized by the European Association of Science Editors, which suggested several areas that would benefit from the contribution of editors of scientific journals (4,5). The responsibility of scientists and scientific journals to communicate evidence-based research data to the public is particularly emphasized.

In the *Croatian Medical Journal*, as one of the journals that published the joint editorial, we stress the need for additional discussion on the global aspects of the environmental crisis. Scientists from different fields are invited to think outside the box and devise possible solutions for improving the damage to Earth’s systems. To quote famous physicist and Nobel Prize winner Richard D. Feynman: “If our small minds, for some convenience, divide…. this universe, into parts – physics, biology, geology, astronomy, psychology, and so on – remember that nature does not know it!”. In other words, input from experts from every walk of science is required to produce solutions for the climate crisis.

The first quarter of the 21st century will be remembered as a period of dramatic climate change and the COVID-19 pandemic. Climate change has had a domino effect on ecosystems, human health, societies, economies, and justice. More and more people are becoming aware of the problem, but only systemic global efforts can bring about real transformation. We are witnessing great solidarity at times of crisis, but also tremendous irrational fears and distrust in science. Anti-science trends are especially embodied in the anti-vaccination movement, which completely rejects the accomplishments of modern medicine. The initiative calling for urgent actions did not only unite medical journals in social activism, but reminded us about our huge responsibilities – to deliver verified scientific information to scientists, communicate research advances to the general public, and safeguard evidence-based science.

Trust in science needs to be reestablished, and the goal of scientific journals is to be even more insistent on all principles of scientific scrutiny. This means that our editorial policy should always uphold strict and unbiased peer-review process, thus granting science the authority that it so desperately needs.
